# Development of a cornea-equivalent phantom in the terahertz frequency region for 3D temperature rise assessment

**DOI:** 10.1038/s41598-025-99950-5

**Published:** 2025-05-16

**Authors:** Shota Yamazaki, Maya Mizuno, Tomoaki Nagaoka

**Affiliations:** National Institute of Information and Communications Technology, Koganei, Tokyo 184-8795 Japan

**Keywords:** Terahertz wave, Cornea-equivalent phantom, 3D temperature measurement, Biological models, Terahertz optics

## Abstract

As the next generation of mobile communication systems (Beyond 5G/6G) is expected to extend into the terahertz (THz) frequency region, it is essential to ensure human safety from electromagnetic waves. For this purpose, the evaluation of the temperature rise caused by the electromagnetic wave energy absorbed by the human body is required. However, there have been no reports on the development of phantoms with dielectric properties equivalent to biological tissues that can be used to measure temperature rise in the THz frequency region by experimental methods. Therefore, in this study, a glycerin-based semisolid phantom was developed to match the dielectric properties of the cornea between 0.1 and 0.6 THz; the cornea is one of the most important tissues for exposure assessment in the THz frequency region. In addition, fluorescent thermoprobes were included in the phantom to measure the temperature rise induced by the absorption of THz waves. The results show that temperature measurements using confocal laser microscopy can be used to obtain temperature distributions in the phantom with a high spatial resolution [2 µm in the transverse direction (xy) and 20 µm in the axial direction (z)] and a high-temperature resolution (0.04 °C).

## Introduction

The recent advancement of technologies in the terahertz (THz) frequency region has sparked a surge in potential applications across diverse fields including chemical sensing^1,2^, security imaging, motion sensing^3–6^, and telecommunications^7–12^. For instance, acquiring precise positioning information in radars essential for autonomous driving and motion sensors is actively underway. The utilization of the THz frequency region is also under consideration in the realm of wireless technology, known as Beyond 5G/6G, aiming for practical implementation in the 2030s. In the coming decades, these technologies using the THz frequency region are anticipated to be applied in practice as an integral element of everyday life. From these points of view, ensuring the safety of humans exposed to electromagnetic waves in the THz region is imperative^13^.

The guidelines of the International Commission on Non-Ionizing Radiation Protection for radio frequencies (ICNIRP, 2020) and laser radiation (ICNIRP, 2013) have already incorporated exposure for the region of THz frequencies^14,15^. At frequencies above 100 kHz, thermal effects are dominant, so guidelines are established on the basis of the increase in internal body temperature owing to the absorption of electromagnetic energy^16,17^. For the investigation of thermal effects, it is essential to evaluate the level of irradiation to tissues, calculate the temperature elevation in tissues^18^, and explore the exposure threshold for tissue damage^19^. Furthermore, the investigated data should be analyzed in detail through computational dosimetry^20^. To achieve highly accurate dosimetry, the dielectric properties of biological tissues are crucial. The dielectric properties are also necessary for creating biological tissue-equivalent phantoms, which are mainly used for dosimetry. The key features of tissue-equivalent phantoms include dielectric properties that can be adjusted by altering component materials, ease of manufacturing into arbitrary shapes, use of inexpensive and readily available ingredients, and their ability to maintain shape without a shell^21,22^. For example, glycerin-based phantoms can replicate high- and low-water-content organs and are mainly used in the GHz frequency range^23^. Most studies on phantoms have targeted frequencies around the GHz region owing to their relevance in current wireless technologies. On the other hand, the phantoms required for evaluating the interaction between the human body and an electromagnetic field (EMF) at higher frequencies remain poorly developed. To develop phantoms that can be employed for the safety assessment of Beyond 5G/6G, the dielectric properties of biological tissues in the THz frequency region are indispensable. In particular, the cornea of the eyeball and the epidermal layer of the skin are major EMF-exposed tissues, and accurately imitating these tissues in phantoms is crucial.

Recently, we have reported the dielectric properties of the epidermis at frequencies up to 0.6 THz, measured by attenuated total reflection-type THz time-domain (THz TD-ATR) spectroscopy^24,25^. In this study, we investigated the dielectric properties of porcine cornea in the 0.1–0.6 THz range. Referring to the obtained dielectric properties of the cornea, we developed a cornea-equivalent glycerin-based phantom. Furthermore, we enhanced the phantom for noninvasive three-dimensional (3D) temperature measurements with high spatial and temperature resolution by incorporating a fluorescent thermoprobe.

## Results

### Manipulation of dielectric properties of semisolid phantom

**Fig. 1:**
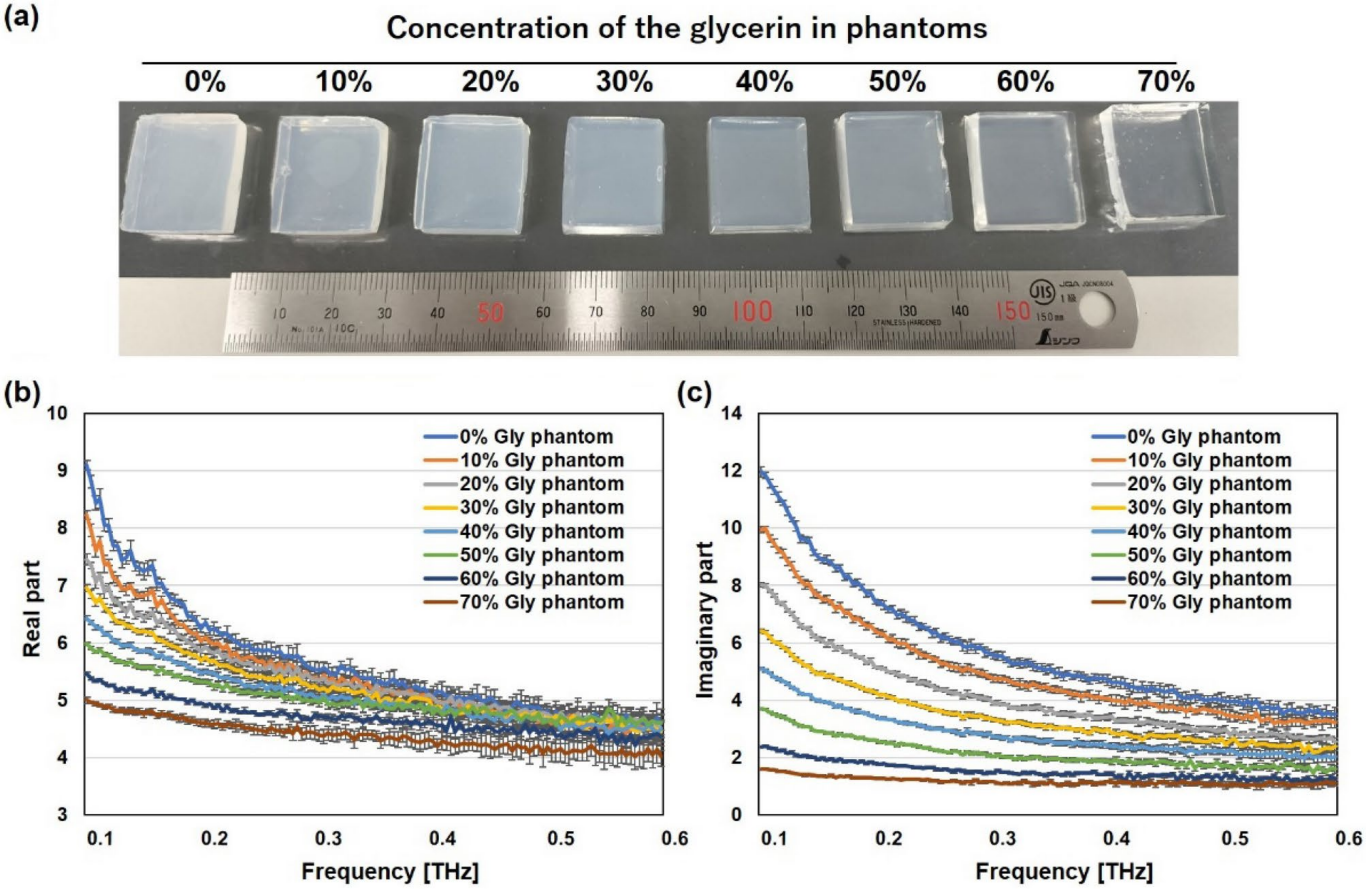
**a** Visible light images of glycerin-based phantoms and the **b** real and **c** imaginary parts of dielectric permittivities at frequencies from 0.1 to 0.6 THz. The mean values and standard deviations are shown (n = 10).

The main factor contributing to the dielectric properties of biological tissues at the THz region is water content^25–27^ In this study, we employed glycerin, physiological saline solution, and agar for adjusting the semisolid phantom at the THz frequency region, because these materials are commonly used for producing the phantom in the GHz region^22^. Figure 1 shows visible light images and the complex relative permittivities of glycerin-based phantoms. Although the permeability of visible light is slightly different in each phantom, the phantom solidity is sufficiently hard to be used in exposure assessment as a semisolid phantom (Fig. 1a). Glycerin was used as the material to regulate water content, and owing to its moisturizing effect, the dielectric properties and dimensions of the phantom remained unchanged for one hour. These phantoms were put on the sample stage for THz TD-ATR spectroscopy and their dielectric properties were measured. The mean values of the real and imaginary parts of dielectric permittivities at frequencies from 0.1 to 0.6 THz, with a standard deviation of 10 samples, are shown in Fig. 1b and c. As a result, we confirmed that glycerin-concentration-dependent changes in water content can regulate the complex relative permittivities in the THz frequency region.

### Dielectric properties of cornea and phantom

**Fig. 2:**
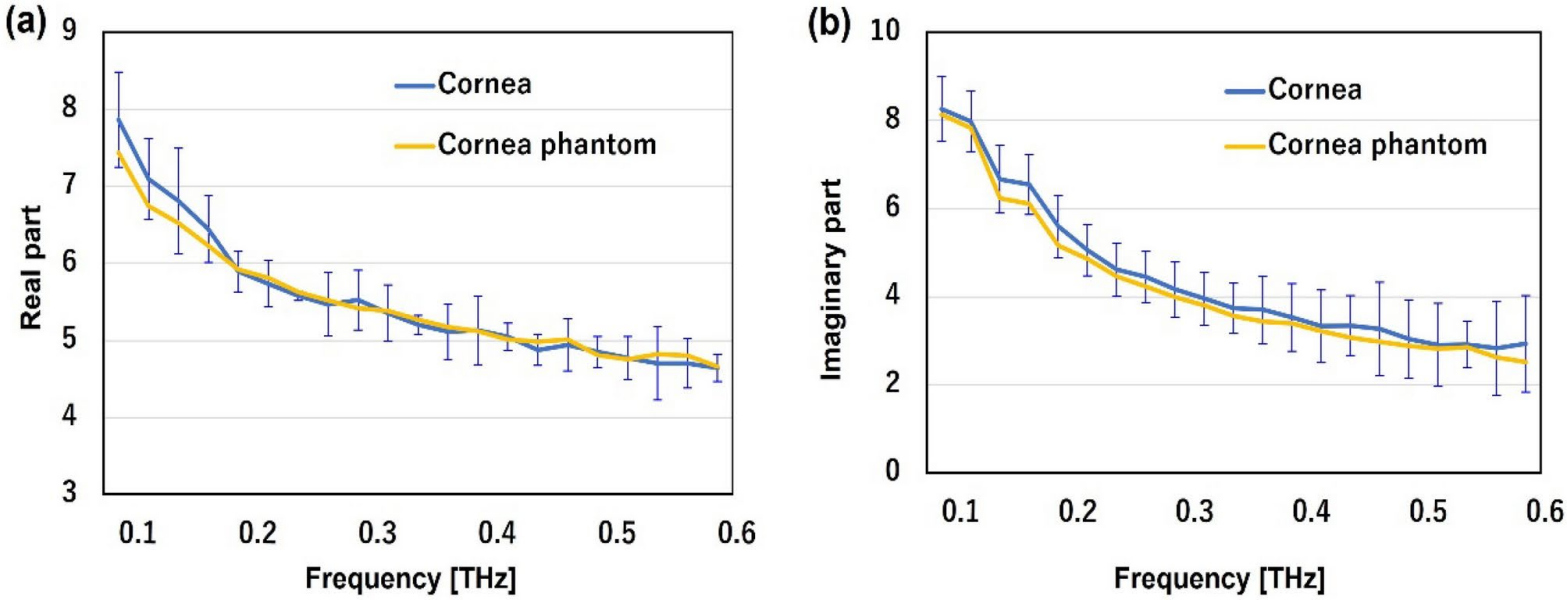
Real and imaginary parts of dielectric permittivities of cornea (blue) and cornea phantom (yellow) at frequencies from 0.1 to 0.6 THz (**a** and **b**). The mean values and S.D. of the cornea are shown (n = 4). S.D. values of the cornea phantom are too small to be seen.

Porcine cornea was used for the measurement of complex relative permittivity because the properties of pigs are physically similar to those of humans^28^. The eyeballs were purchased from DARD. Co. Ltd. and cornea was isolated using a surgical knife. Figure 2 shows the real and imaginary parts of the complex relative permittivity of the cornea and the imitating phantom. Here, mean values with a standard deviation of four corneas were represented. The maximum errors of the cornea are 23% (real part) and 37% (imaginary part). Referring to the glycerin-concentration-dependent changes of the complex relative permittivities shown in Fig. 1, we made a cornea-equivalent phantom composed of 79.5% saline solution, 20% glycerin, and 0.5% agar. The complex relative permittivities of both the real and imaginary parts for the cornea (blue) and the cornea-equivalent phantom (yellow) showed good agreement. Moreover, the values of the cornea-equivalent phantom were all within the range of errors of the cornea across the 0.1–0.6 THz frequency region. This result indicated that the glycerin-based phantom can reproduce the dielectric properties of the cornea at a broad range of THz frequencies.

### Effect of fluorescent thermoprobe on the dielectric permittivities

**Fig. 3:**
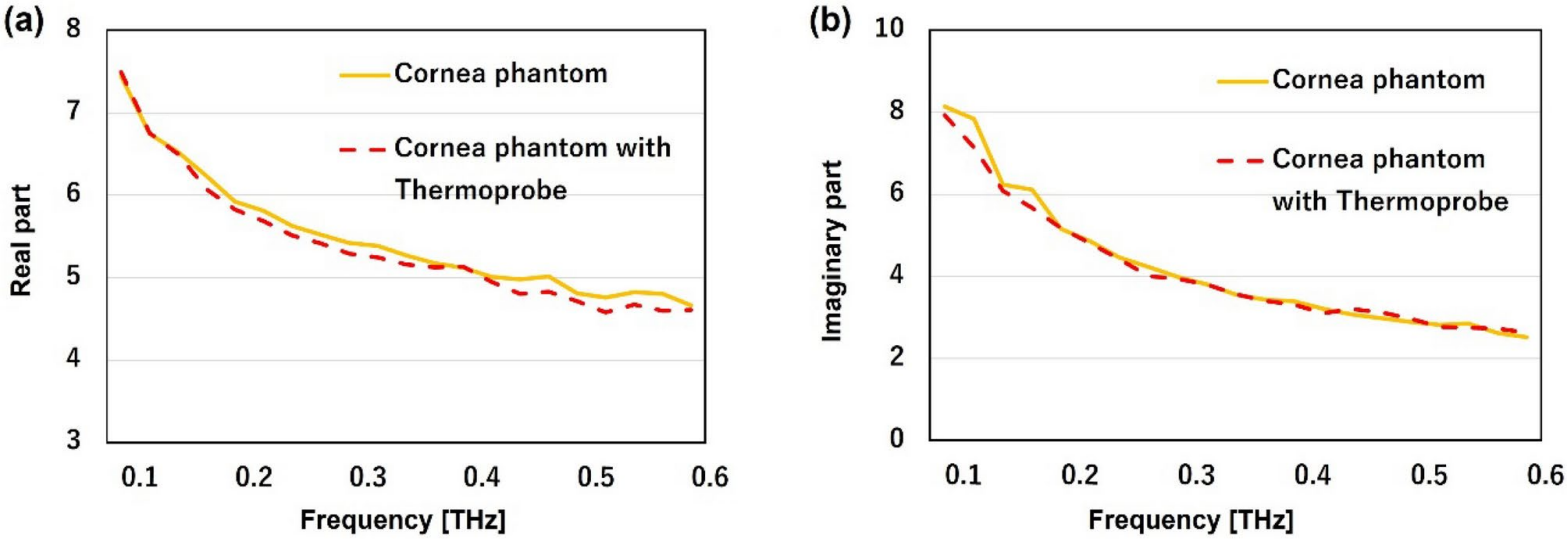
Mean values of real part **a** and imaginary part **b** of dielectric permittivities of cornea phantom (yellow) and cornea phantom with thermoprobe (red) at frequencies from 0.1 to 0.6 THz (n = 4).

The high absorbance of liquid water in the THz frequency range restricts the penetration depth of THz waves within biological tissues to several hundred micrometers. In this transmittance process, the absorbed power leads to temperature increases on the surface of the human body within a depth of a few hundred micrometers. However, the spatial resolution of thermometers, such as fiber optic thermometers, is about 1 mm, limited by sensor size, making it difficult to measure temperature changes induced by THz waves. Furthermore, conventional methods such as the thermal camera lack the capability to perform a depth dimensional analysis of temperature distribution^29^. To achieve 3D temperature measurement in lateral (x, y) and axial (z) directions inside phantoms, we employed a fluorescent thermoprobe that has been used for visualizing temperature distribution in cultured cells with high-temperature resolution (0.01 °C) and high spatial resolution at the molecular scale^30^. Figure 3a and b show the complex relative permittivities of the cornea-equivalent phantoms with and without the thermoprobe. Deviations from the mean values of phantoms with and without the thermoprobe mostly remained within 2% (real part) and 1% (imaginary part) across the 0.1–0.6 THz frequency range. Most importantly, the values of the phantom with the thermoprobe were all within the error range of the values of the cornea. These results indicate that we can ignore the effect of thermoprobes on the complex relative permittivities in this frequency region.

### Function of the fluorescent thermoprobe in the cornea-equivalent phantom

**Fig. 4:**
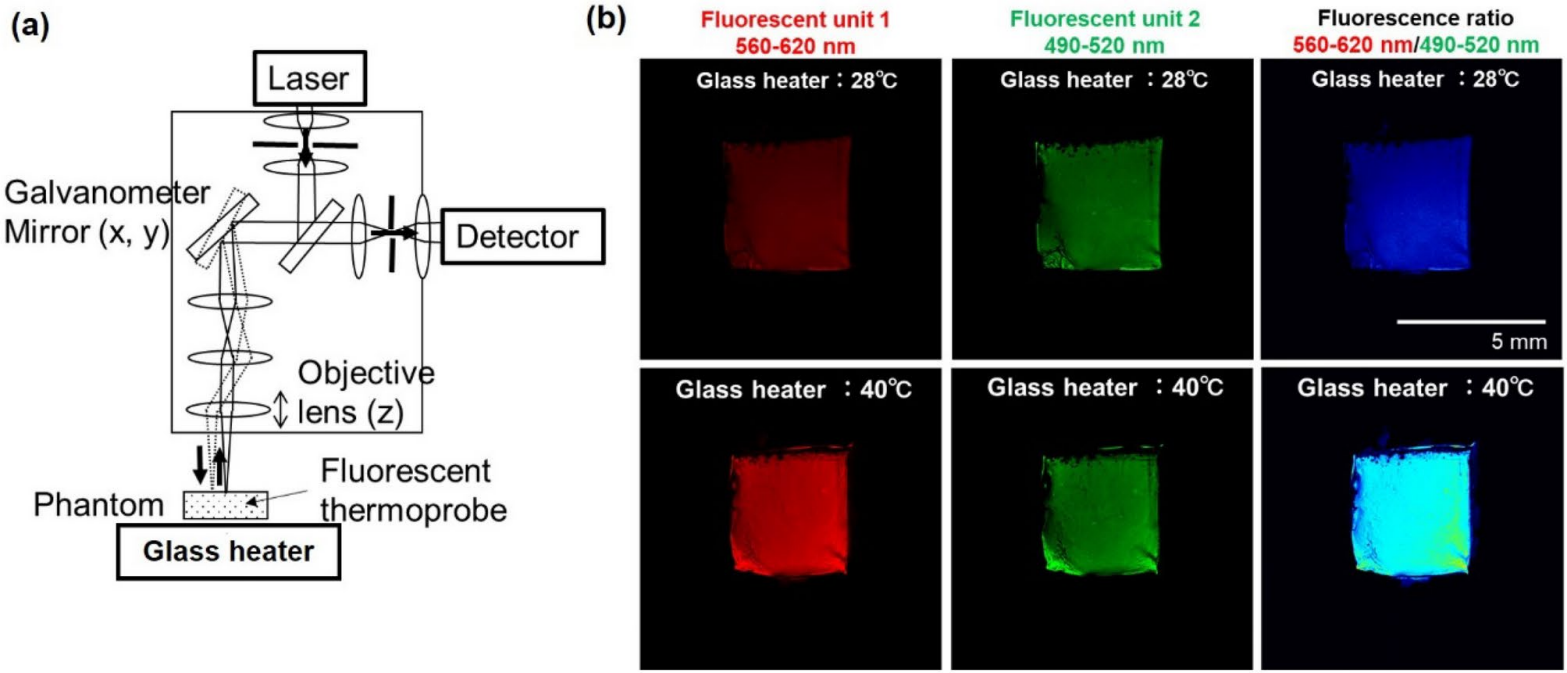
**a** Schematic diagram of optical configuration for confocal laser scanning microscopy and **b** change of fluorescent thermoprobe in a cornea-equivalent phantom. Scale bars represent 5 mm.

The fluorescent thermoprobe, originally developed for measuring temperature inside cells, was assessed for its functionality when embedded in a phantom semisolidified with agar. The thermoprobe is composed of three units: thermosensitive units, environment-sensitive fluorophores (fluorescent unit 1, excitation: 488 nm/emission: 560–620 nm) and reference fluorophores (fluorescent unit 2, excitation: 488 nm/emission: 490–520 nm). The intensity of fluorescent unit 1 changes depending on temperature. Notably, this intensity not only varies with temperature but also depends on the concentration of the thermoprobe. Achieving uniform dispersion of the thermoprobe at a consistent concentration within the phantom is difficult. Furthermore, shrinking the phantom can alter the intensity of fluorescent unit 1 owing to concentration changes. To mitigate this issue, fluorescent unit 2, maintained at the same concentration of fluorescent unit 1, is included in the thermoprobe to compensate for concentration effects. The fluorescence intensity of fluorescent unit 2 remains constant. Therefore, by using the relative intensities of fluorescent unit 1 and fluorescent unit 2, changes in the intensity owing to temperature can be evaluated without being affected by variations in thermoprobe concentration. A schematic view of the basic construction of the temperature visualization system using confocal laser scanning microscopy is shown in Fig. 4a. The focus of the laser beam was manipulated in the xy direction using a galvanometer mirror. Additionally, the focal plane along the z-axis was adjusted by moving the objective lens up and down^31^. The cornea-equivalent phantom with fluorescent thermoprobes was put on the thermoplate to control the temperature. Fluorescent images of fluorescent unit 1 and fluorescent unit 2 at the phantom surface in contact with the thermoplate were obtained by confocal laser scanning microscopy (FV3000, Olympus). Figure 4b shows the fluorescent images of the semisolidified cornea-equivalent phantom heated at different temperatures. Compared with the case of heating to 28 °C, the intensity of fluorescent unit 1 (emission: 560–620 nm) increased in the phantom heated to 40 °C. On the other hand, the intensity of fluorescent unit 2 (emission: 490–520 nm) used as a reference was changed by less than 1% between 40 and 28 °C. The fluorescence ratio of fluorescent units 1 and 2 was represented by color. The lower left end of the phantom was aggregated by heating to 40 °C. This deformation increased the concentration of the thermoprobe, resulting in a higher fluorescence in unit 1. However, since the fluorescence intensity of the reference fluorescent unit 2 also increased, we confirmed that by taking the ratio, we can evaluate only the temperature rise, ignoring the influence of concentration. The temperature-dependent change in the fluorescence intensity ratio of fluorescent units 1 and 2 indicates that this ratio serves as an appropriate marker for assessing temperature in agar-solidified phantoms.

### Measurements of the fluorescence spectra of the fluorescent thermoprobe

**Fig. 5:**
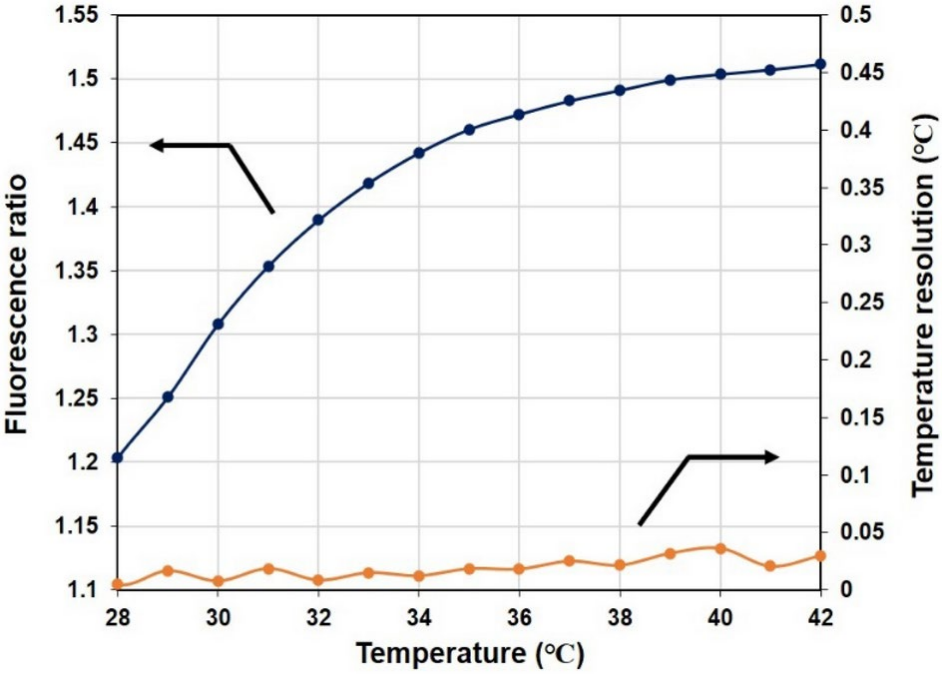
Fluorescence ratio (left axis) and temperature resolution (right axis) of fluorescence thermoprobe in the cornea-equivalent phantom. The error bars for the fluorescence response representing the S.D. (n = 6) are too small to be visualized.

Next, we confirmed the corresponding relationship between the fluorescent ratio of the thermoprobe and temperature of the cornea-equivalent phantom. Figure 5 shows the calibration curve of the temperature-dependent fluorescent ratio (left axis). The fluorescence ratios were examined at the center region (100 μm × 100 μm) of the cornea-equivalent phantom, at which the phantom was in direct contact with the thermoplate regulated at each temperature. The phantom temperature was measured with a k-type coupler (HD-1200 K, Anritsu Meter) with a measurement accuracy of ± 0.2 °C for calibration. The error bars for the fluorescence ratio, representing the standard deviation (n = 6), are too small to be visualized. The calibration curve showed that the change of the fluorescent ratio was strongly correlated with the temperature as the temperature varied from 28 to 42 °C, and the fluorescent thermoprobe was an appropriate marker for assessing the temperature in the cornea-equivalent phantom.

The right axis of Fig. 5 shows the temperature resolution. The temperature resolution (δT) of the fluorescent thermoprobe was evaluated using the following equation^31^:

$$\delta T = \left( {\frac{\partial T}{{\partial F_{ratio} }}} \right)\delta F_{ratio} ,$$

where ∂T/∂F_ratio_ and δF_ratio_ represent the inverse of the slope of the fluorescence ratio–temperature diagram and the standard deviation of the fluorescence ratio, respectively. The standard deviation was obtained for six measurements of one sample at each temperature. The temperature resolution of the fluorescent thermoprobe for thermometry of the cornea-equivalent phantom was determined to be 0.01–0.04 °C in the temperature range between 28 and 42 °C. This temperature resolution was comparable to that of the previously reported fluorescent thermoprobe^30^.

### Analysis of spatial resolution

**Fig. 6:**
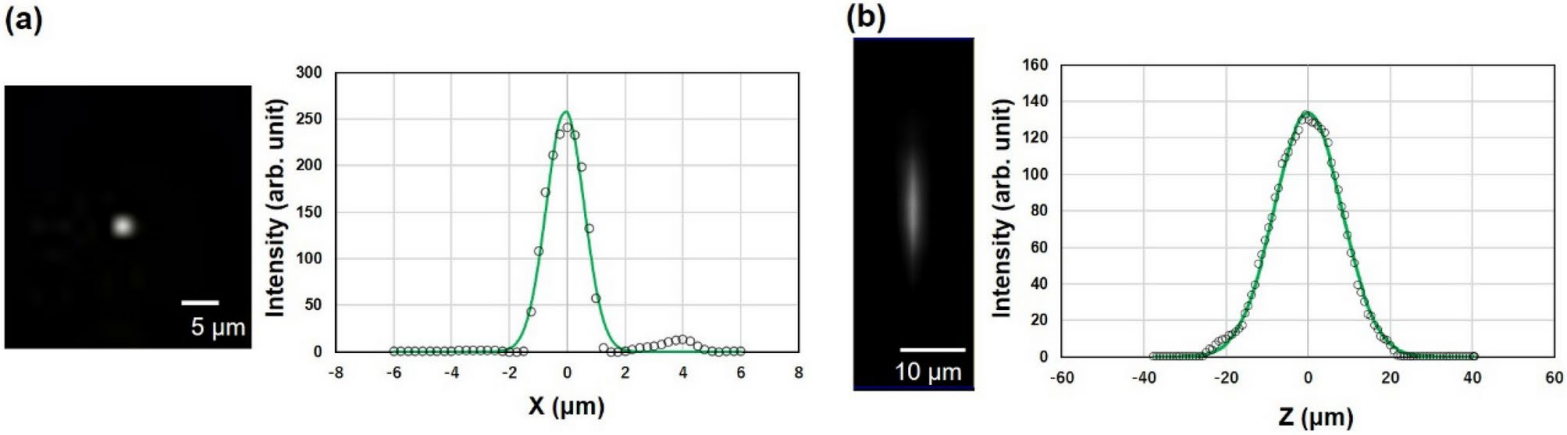
Analysis of the spatial resolution. **a** Lateral and **b** axial spatial resolution examined by measuring point-spread-function full width at half-maximum (FWHM) and Gaussian fitting (green line). Fluorescent beads with 1 µm diameter (F8823, ThermoFisher) embedded in the phantom were employed for the analysis of FWHM.

The advantage of confocal laser scanning microscopy is the higher spatial resolution of micrometer-scale order. Therefore, we analyzed the spatial resolution of the fluorescent thermoprobe in the cornea-equivalent phantom. Fluorescent beads with 1 µm diameter (F8823, ThermoFisher) embedded in the phantom were employed for the analysis of the point-spread-function full width at half-maximum (FWHM), which indicates the actual spatial resolution. Figure 6a shows the intensity profile of a fluorescent bead in a lateral scan (XY direction) with a UPlanFL N 10 × objective lens (Olympus) and the Gaussian fitting line (green). The fluorescence intensity profile shows an FWHM of 2 μm. Figure 6b shows the intensity profile of a fluorescent bead in an axial scan (Z direction) with a 1 μm step and its Gaussian fitting line. The measured axial resolution was 20 μm.

### Image of temperature distribution inside cornea-equivalent phantom

**Fig. 7.**
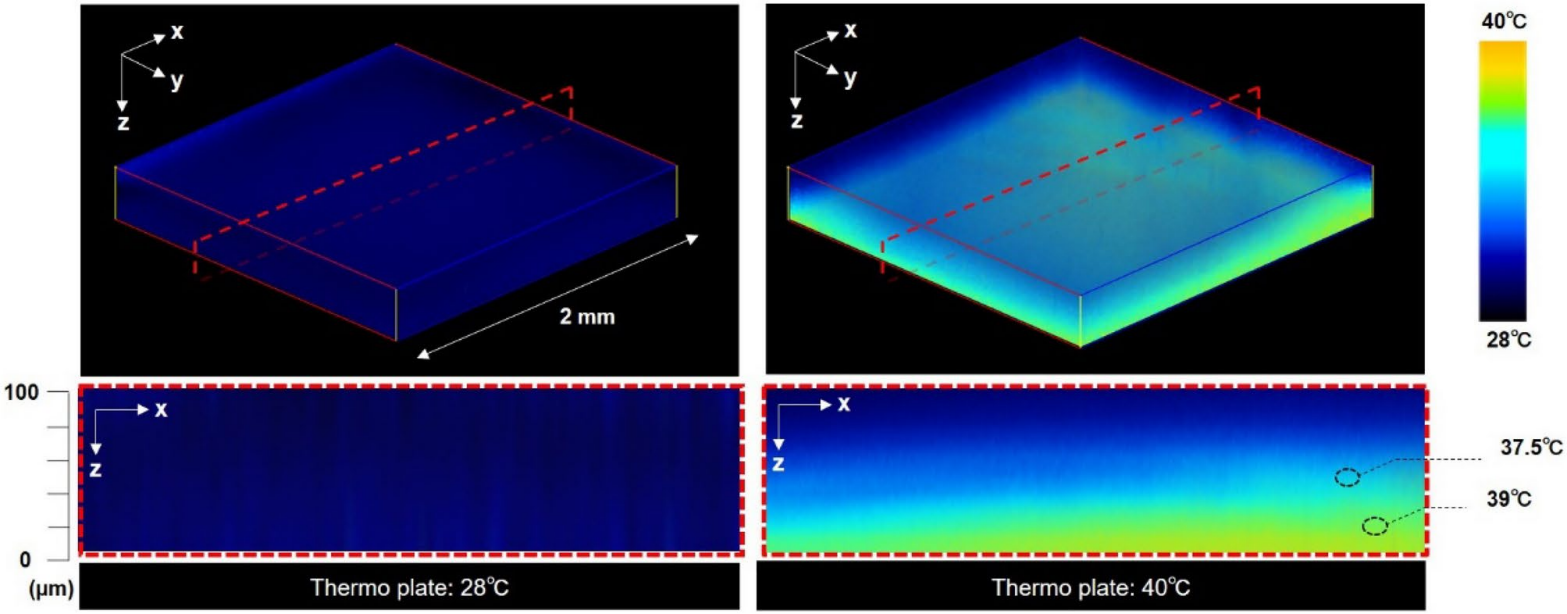
3D and cross-sectional images of temperature distribution inside the cornea-equivalent phantom. The intensities of thermoprobes were scanned across the surface in contact with the thermoplate with 4 μm steps, and a 3D image was reconstructed from the corresponding sectional images within a 100-μm-thick region.

Figure 7 shows 3D and cross-sectional images of the temperature distribution inside the cornea-equivalent phantom. The phantom put on the thermoplate was heated for 15 min at 28 °C or 40 °C. After 15 min of heating, the temperature reached a thermal equilibrium state. The 3D image was generated by acquiring cross-sectional images in the planar (xy) direction at intervals of 4 µm along the depth (z) direction, followed by their reconstruction. A 3D image was reconstructed from the corresponding 26 sectioned images. The time required to capture a single tomographic image is approximately 1 s. The varying colors across the image represent different temperatures within a depth of 100 μm in the phantom heated to 40 °C. For example, at a distance of about 20 μm from the plate, the temperature was 39 °C, and at a further distance of about 40 μm , the temperature was 37.5 °C. This high-resolution mapping offers insights into the precise temperature changes and distribution within the phantom, crucial for simulating and understanding thermal responses to the exposure to THz waves.

## Discussion

The phantom has been primarily used for dosimetry and evaluating antenna performance during research and development processes^21–23^. Its features include easy adjustment of dielectric properties, ease of manufacturing into arbitrary shapes, use of inexpensive and widely available ingredients, and the ability to maintain its shape without a shell. In this study, we aimed to create a phantom for the THz frequency region and found that glycerin is a suitable material for modulating water content and preventing the evaporation of moisture. As shown in Fig. 1, glycerin-based phantoms exhibit adjustable complex relative permittivities in the THz region, making them useful for simulating various biological tissues with different dielectric properties^33^. On the basis of these results, we developed a glycerin-based solid phantom with the complex relative permittivity of porcine cornea in the THz region. The complex relative permittivities of both the real and imaginary parts of the cornea and the cornea-equivalent phantom showed good agreement, with the values of the cornea-equivalent phantom falling within the error range of values of the cornea across the 0.1–0.6 THz frequency region. This outcome suggests that the glycerin-based phantom can replicate the dielectric properties of the cornea over a wide range of THz frequencies.

Recently, the temperature rise in phantoms has been mainly analyzed by simulations^22,34^. In order to validate the simulation results, simulated temperature changes must be compared with experimental measurements. In addition, considering the region where THz waves are absorbed, information on temperature changes inside tissues is important. In fact, the evaluation of temperature deep inside the phantom is crucial because there are structures within tissues, such as the corneal endothelium, that do not recover once damaged^35^. However, the thermocamera that is generally used for temperature measurement does not have sufficient spatial resolution in the depth direction. For temperature measurement inside materials, fiber-optic thermometers are useful; however, they have disadvantages such as the need to insert the sensor into the sample and the ability to measure only a single point. Thus far, a method for 3D temperature measurement inside the phantom has not yet been developed. Therefore, we improved the phantom used in a temperature measurement system by incorporating a fluorescent thermoprobe. As shown in Fig. 7, we confirmed that the 3D temperature distribution can be observed with excellent spatial precision that achieves 2 μm in the lateral (xy) directions and 20 μm in the axial (z) direction. This is coupled with a high-temperature resolution better than 0.04 °C.

## Conclusion

In summary, in our research, we not only established the glycerin-based phantom as a valuable tool for THz exposure assessments but also demonstrated its potential for high-precision temperature measurements in this frequency range. These findings contribute to the broader understanding of electromagnetic field safety, especially in the context of advancing THz technologies, and pave the way for future applications in fields such as medical diagnostics and wireless communication.

## Methods

### Spectroscopy system

THz TD-ATR spectroscopy (TAS7500TS, ADVANTEST Co.)^24,25^ is employed to determine complex refractive indices and to calculate the complex dielectric constants in the 0.1–0.6 THz region of porcine cornea and phantoms. Because of the difficulty of obtaining human corneas, porcine corneas are often substituted as models for cornea research. In fact, a study of the cornea in 45 species has revealed that the structures and compositions are almost constant, indicating that mammalian corneas have similar dielectric properties^28^. Thus, tissues of experimental animals such as pigs or rabbits are widely used as human substitutes. In the optical configuration for ATR spectroscopy, the THz pulses are totally reflected from the interface between air and the silicon prism, and an evanescent wave interacts with the sample placed on the top of the prism in a region several hundred micrometers thick. THz time-domain waveforms with and without a sample were detected and Fourier-transformed into an amplitude E(ω) and a phase spectrum φ(ω). Subsequently, the complex dielectric constant ε(ω) was calculated from E(ω) and φ(ω) using Fresnel’s equation. All samples were put on the silicon stage and measured using the THz TD-ATR spectrometer.

### Preparation of the phantoms

The phantoms were composed of physiological saline solution, glycerin, and agar. These materials are used generally for producing the phantom for the GHz region^22^. The physiological saline solution and glycerin were dispensed using a micropipette and mixed in a beaker glass. Then, 2% agar was added and stirred well using a spatula. The beaker glass was wrapped, and the solution was heated to 100 °C in a microwave oven and stirred. This process was continued until the agar was completely dissolved. After its temperature returned to 45 °C with stirring, the solution was poured into a cube mold and left at room temperature for 30 min. 200 µg of fluorescence thermoprobe powder was reconstituted in 20 µl of ultrapure water and incubated at 4 °C for at least 12 h. 10 µl of the stock solution incubated at 4 °C was added to a 2 ml phantom solution after being heated in a microwave oven and poured into a cube mold to solidify. The concentration of the fluorescence thermoprobe was determined after confirming that the dielectric properties were not affected and the intensity changed depending on the temperature.

### Measurement of the semisolid phantom temperature using fluorescent polymeric thermometers

The temperature in the semisolid phantom was measured using a Cellular Thermoprobe for Fluorescence Ratio (#FDV-0005, Funakoshi Co.)^30^. The fluorescence thermoprobe is a random copolymer composed of distinct units: thermosensitive units, environment-sensitive fluorophores (fluorescent unit 1, Ex: 458 nm/Em: 560–620 nm) and reference fluorophores (fluorescent unit 2, Ex: 458 nm/Em: 490–520 nm). At low temperatures, the thermosensitive unit in the fluorescence thermoprobe assumes an extended structure such that neighboring water molecules can quench the water-sensitive fluorescent unit 1. At higher temperatures, the thermosensitive unit shrinks because of hydrophobic interactions among the thermosensitive units, resulting in the release of water molecules and strong fluorescence from fluorescent unit 1. In addition, the fluorescence intensity of fluorescent unit 2 is insensitive to the temperature-dependent changes in the thermosensitive unit, and its constant fluorescence is utilized as a reference signal. The temperature-dependent fluorescence intensities of the fluorescence thermoprobes enable us to perform highly sensitive and practical ratiometric temperature sensing.

### Fluorescence imaging of phantoms

Phantoms treated with the fluorescence thermoprobe were observed using a confocal microscope (FV3000, Olympus) with a PLAPON1.25 × lens (Olympus, N.A. 0.04) and UPlanFL N 10 × lens (Olympus, N.A. 0.3). The emission from the thermometer between 490 and 620 nm was collected under excitation at 488 nm. The resulting fluorescence images were analyzed using cellSens (Olympus). The temperature of the culture medium was controlled with a Thermo Plate (TOKAI HIT).

## Data Availability

Data availability statement The data supporting the findings of this study are available from the corresponding author upon reasonable request.
